# Copper-related genes predict prognosis and characteristics of breast cancer

**DOI:** 10.3389/fimmu.2023.1145080

**Published:** 2023-04-27

**Authors:** Yi Liu, Jiandong Wang, Mengxi Jiang

**Affiliations:** ^1^ Department of Pharmacology, School of Basic Medical Sciences, Capital Medical University, Beijing, China; ^2^ Department of General Surgery, The First Medical Center, Chinese People's Liberation Army (PLA) General Hospital, Beijing, China; ^3^ Advanced Innovation Center for Human Brain Protection, Capital Medical University, Beijing, China

**Keywords:** breast cancer, copper metabolism, cuproptosis, prognosis, characteristics

## Abstract

**Background:**

The role of copper in cancer treatment is multifaceted, with copper homeostasis-related genes associated with both breast cancer prognosis and chemotherapy resistance. Interestingly, both elimination and overload of copper have been reported to have therapeutic potential in cancer treatment. Despite these findings, the exact relationship between copper homeostasis and cancer development remains unclear, and further investigation is needed to clarify this complexity.

**Methods:**

The pan-cancer gene expression and immune infiltration analysis were performed using the Cancer Genome Atlas Program (TCGA) dataset. The R software packages were employed to analyze the expression and mutation status of breast cancer samples. After constructing a prognosis model to separate breast cancer samples by LASSO-Cox regression, we examined the immune statement, survival status, drug sensitivity and metabolic characteristics of the high- and low-copper related genes scoring groups. We also studied the expression of the constructed genes using the human protein atlas database and analyzed their related pathways. Finally, copper staining was performed with the clinical sample to investigate the distribution of copper in breast cancer tissue and paracancerous tissue.

**Results:**

Pan-cancer analysis showed that copper-related genes are associated with breast cancer, and the immune infiltration profile of breast cancer samples is significantly different from that of other cancers. The essential copper-related genes of LASSO-Cox regression were ATP7B (ATPase Copper Transporting Beta) and DLAT (Dihydrolipoamide S-Acetyltransferase), whose associated genes were enriched in the cell cycle pathway. The low-copper related genes scoring group presented higher levels of immune activation, better probabilities of survival, enrichment in pathways related to pyruvate metabolism and apoptosis, and higher sensitivity to chemotherapy drugs. Immunohistochemistry staining showed high protein expression of ATP7B and DLAT in breast cancer samples. The copper staining showed copper distribution in breast cancer tissue.

**Conclusion:**

This study displayed the potential impacts of copper-related genes on the overall survival, immune infiltration, drug sensitivity and metabolic profile of breast cancer, which could predict patients’ survival and tumor statement. These findings may serve to support future research efforts aiming at improving the management of breast cancer.

## Introduction

Breast cancer has become a significant worldwide health issue, with over two million emerging cases and six hundred thousand death records in 2020 (1, 2). Common treatment options, such as chemotherapy, endocrine therapy, immunotherapy and radiotherapy, do not always provide optimal therapeutic effects to breast cancer patients (3). Therefore, it is important to develop more accurate and effective prognostic models that can effectively characterize and classify the molecular subtypes of breast cancer in order to diagnose, treat and prevent breast cancer in a more precise manner.

Copper is a cofactor for various enzymes and plays a vital role in cellular metabolism and respiration, and disruption of copper homeostasis cause Wilson disease and Menkes disease (4, 5). Copper also contributes to cancer development by enhancing tumor cell proliferation and angiogenesis. Consequently, copper chelator has been applied to inhibit cancer metastasis in clinical trials (6–8). On the contrary, copper overload has been recently proposed to induce lipoylated protein aggregation and cancer cell death (9). Copper homeostasis-related genes have been implicated in breast cancer prognosis and chemotherapy resistance. Studies have shown that breast cancer patients with poor prognoses exhibit higher expression of the copper importer solute carrier family 31 member 1 (SLC31A1) and the copper binding protein ceruloplasmin, which could be utilized as potential prognosis factors (10–12). Decreased expression of the copper exporters ATPase copper transporting α (ATP7A) and ATPase copper transporting β (ATP7B) have been associated with decreased chemotherapy resistance in breast cancer cells (13, 14). It is currently not fully understood how copper metabolism may be involved in breast cancer or the potential mechanisms by which it may influence the development or progression of the disease. Therefore, a comprehensive analysis of the genetic alterations of copper-related genes in tumor tissue could identify molecular targets for future diagnosis and treatments for breast cancer.

Our pan-cancer analysis identified a differential expression pattern of copper-related genes and immune cell infiltration profile in breast cancer. We further investigated the expression and copy number variation (CNV) of copper-related genes in breast cancer and separated breast cancer samples based on the risk score. We then compared the survival status, immune status, drug sensitivity and metabolic pathways of the high- and low-copper related genes scoring groups. Specifically, we analyzed the protein expression, the related genes and the metabolic pathways of the essential copper-related genes, namely ATP7B and DLAT, in breast cancer samples. The clinical sample also confirmed that copper is distributed in breast cancer tissue. In summary, this study may offer valuable insights for identifying potential therapeutic interventions and biomarkers for breast cancer treatment.

## Materials and methods

### Acquisition of copper-related genes and data collection

We collected copper metabolism-related genes from MSigDB (15) and cuproptosis-related genes from literature (9). The 42 copper-related genes are listed in **Table S1**. The transcriptome data and medical information of breast cancer patients were obtained from the Cancer Genome Atlas (TCGA) database (https://www.cancer.gov/tcga). After excluding samples with incomplete transcriptomic and survival data, we obtained a final dataset with 1069 breast cancer samples and 113 paracancerous samples, which were used for the following analysis. The validating datasets were procured from Gene Expression Omnibus (GEO), including GSE96058 with 3273 breast cancer samples (16), GSE18229 with 82 samples of luminal A and HER2-enriched subtypes (17), and GSE58812 with 107 samples of triple-negative breast cancer (18). The data of Infiltration Estimation for all TCGA tumors were obtained from TIMER2.0 (19). Copy number variation landscape was presented by the R package “maftools” (20).

### Heatmap, PPI network, and correlation network

The heatmap was presented by chiplot (https://www.chiplot.online/) and data were collected from TCGA database and Genotype-Tissue Expression (GTEx) based on UCSC XENA platform (21). The PPI network (Protein-Protein Interaction Networks) was created by the STRING database (22) and Cytoscape (23). The degree of cuproptosis and copper metabolism-related genes was calculated by CytoNCA (24). The correlation network was presented by the R package “corrr”.

### Construction and validation of the copper-related genes’ prognostic index

Copper-related genes were analyzed by univariate Cox regression and genes with *p* < 0.05 were integrated into the LASSO-Cox regression *via* 10-fold cross-validation in order to narrow down candidate genes. A prognostic signature was built by multivariate Cox regression, whose predictive capability on overall survival (OS) was analyzed by time-dependent receptor operating characteristic (ROC) curves by using the R package “timeROC” and “ggplot2” (25). The univariate and multivariate Cox regression results were obtained from the online analysis platform ToPP (http://www.biostatistics.online/topp/index.php.) (26).

### Survival analysis

The Kaplan–Meier curve was performed to compare the survival status of the high- and low-copper related genes scoring groups stratified by the risk score of copper-related genes using the R packages “survival”, “survminer” and “ggplot2” (R version 4.1.3). Genes were considered statistically significant at the *p* < 0.05 level.

### Immune profile analysis

In order to identify the immune states and prognostic features of the high- and low-copper related genes scoring groups, we applied CIBERSORT (27) to evaluate and compare the immune composition between the two groups. By Tumor Immune Dysfunction and Exclusion (TIDE) (28), we obtained the MSI (microsatellite instability), Exclusion and Dysfunction to compare the potential of tumor immune escape between the two groups. We calculated the stromal score, immune score, tumor purity and estimated score through the ESTIMATE algorithm (29).

### Immunohistochemical staining of ATP7B and DLAT by the human protein atlas (HPA) database

The gene expression data based on breast cancer clinical specimens were obtained from the HPA database (https://www.proteinatlas.org/). Visualizing data of HPA were presented using the R package “HPAanalyze”.

### GSEA

Gene set enrichment analysis (GSEA) of the high- and low-copper related genes scoring groups was created by the desktop application of GSEA 4.2.3. Pathways were considered statistically enriched at the cut-off point of *p< 0.05* and *FDR* < 0.25 (15).

### Drug sensitivity analysis

Based on the transcriptome data of breast cancer samples, the drug sensitivity was analyzed by the R package “oncoPredict” and the Genomics of Drug Sensitivity in Cancer (GDSC) database (30).

### LinkedOmics analysis

The LinkFinder and LinkInterpreter modules of the LinkedOmics web application were employed to investigate the potential gene regulation network of the signature genes (31). These tools allowed for identifying and analyzing relevant attributes, providing insight into the functional relationships and regulatory mechanisms at play in the network.

### Copper staining of breast cancer samples

Tissue sections were obtained from both cancerous and paracancerous areas of a patient with stage III/IV breast cancer that tested negative for both estrogen receptor (ER) and progesterone receptor (PR). The tissue sections were fixed with 4% formaldehyde (G1101; Servicebio, Wuhan, China) overnight. After dehydration, wax leaching, deparaffinization and rehydration with ethanol and xylene, the slides were stained following the kit manufacturer’s instructions for copper stain (M094; Gefanbio, Shanghai, China) followed by hematoxylin stain (G1004-500ML; Servicebio, Wuhan, China). The histological images of the tissue sections were scanned by a digital slide scanner (Pannoramic scan, Hungary). This study was approved by the ethics committee of the Chinese People's Liberation Army (PLA) General Hospital (No. S2016-055).

### Statistical analysis

The R version 4.1.3 was used to analyze data. The comparative methods of difference between the groups were applied, including Student’s t-test, Wilcoxon test, Kruskal-Wallis, and Log-Rank test for survival analysis. The asterisks symbolized the statistical *p* value (^*^p < 0.05; ^**^p < 0.01; ^***^p < 0.001, ^****^p< 0.0001).

## Results

### The pan-cancer expression patterns of the copper-related genes and the pan-cancer immune statement

**Figure 1:**
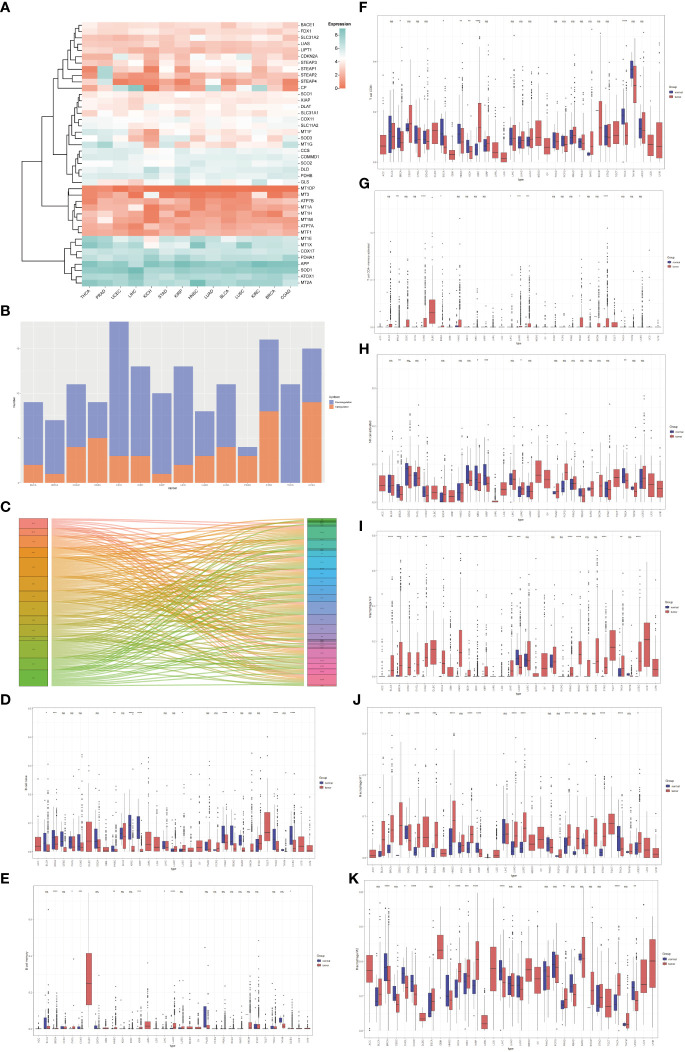
The pan-cancer analysis of copper-related genes. **(A)** Heatmap of copper-related genes showed different expression patterns across different types of cancers. **(B)** A stacked bar chart of copper-related genes in different types of cancer samples showed the number of differentially expressed genes. The red and blue colors represented upregulated and downregulated genes, respectively. **(C)** The Sankey diagram of differentially expressed copper-related genes across different cancer types. **(D–K)** Box plot comparison of the abundance of naive B cells **(D)**, memory B cells **(E)**, CD8^+^ T Cells **(F)**, memory CD4^+^ T cells **(G)**, activated NK cells **(H)**, M0 macrophages **(I)**, M1 macrophages **(J)**, and M2 macrophages **(K)** in different types of cancers compared with paired non-tumor samples. (*p < 0.05; **p < 0.01; ***p < 0.001, ****p< 0.0001,NS: no significance).

Based on the Molecular Signatures Database (MsigDB) (15) and the recent cuproptosis literature (9), we selected 42 copper-related genes for analysis (**Table S1**). The expression of copper-related genes in 14 cancer types was examined and demonstrated by a heatmap (**Figure 1A**). The stacked bar chart showed differentially expressed copper-related genes in different cancer types (**Figure 1B**). The Sankey diagram showed the log2 fold change (tumor vs. non-tumor sample) of differentially expressed copper-related genes across different cancer types (**Figure 1C**). These results demonstrated the dysregulation of copper-related genes in breast cancer and other cancer types. To further identify the immune profile of different types of cancer, we generated the boxplot to compare the immune cells’ infiltration profile in tumor samples and their paired non-tumor samples. The boxplot showed the different immune cells statement of tumor samples, demonstrating that the enrichment of naive B cells (**Figure 1D**), memory B cells (**Figure 1E**), CD8^+^ T Cells (**Figure 1F**), activated memory CD4^+^T Cells (**Figure 1G**), activated NK cells (**Figure 1H**), M0 macrophages (**Figure 1I**), M1 macrophages (**Figure 1J**) and M2 macrophages (**Figure 1K**) was significantly changed in many cancer types, especially in breast cancer samples.

### The expression and genetic variation profile of copper-related genes in breast cancer samples

**Figure 2:**
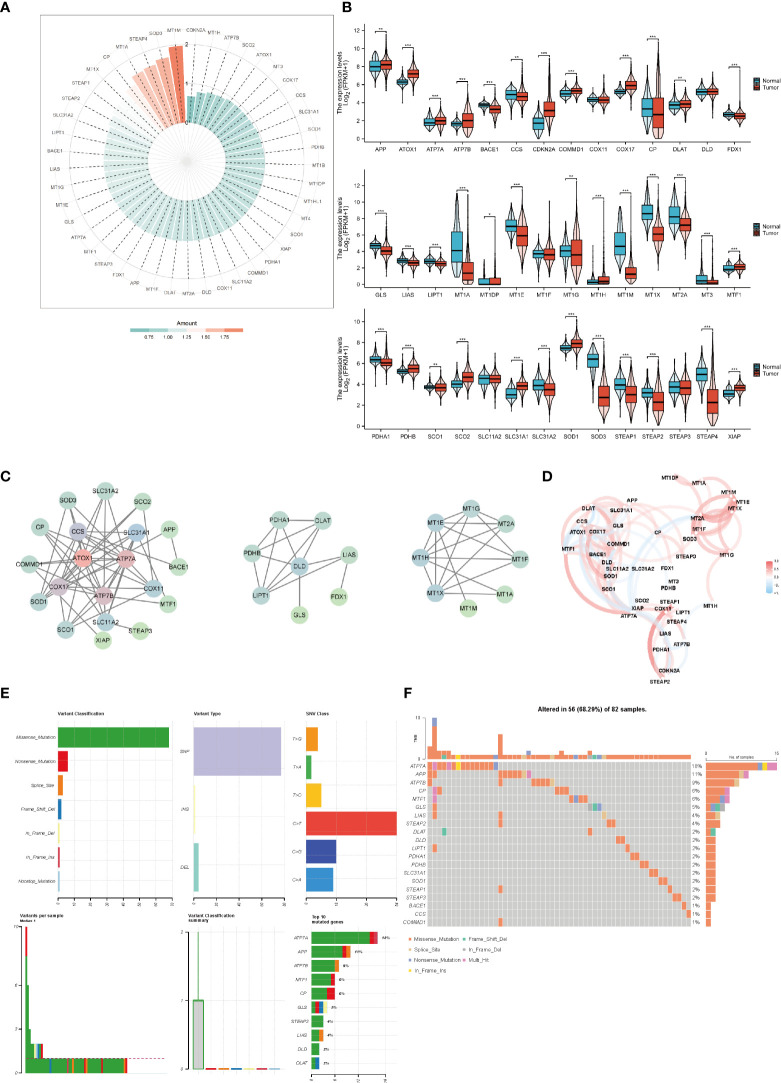
The expression and genetic variation of copper-related genes in breast cancer samples. Heatmap **(A)** and box plots **(B)** of differentially expressed copper-related genes in breast cancer samples. **(C)** PPI network of copper-related genes. **(D)** Correlation of copper-related genes in breast cancer samples. CNV, mutation frequency **(E)** and classification **(F)** of copper-related genes in breast cancer samples. (^*^p < 0.05; ^**^p < 0.01; ^***^p < 0.001).

We analyzed the expression of copper-related genes in breast cancer and non-tumor samples, which verified that breast cancer samples had dysregulation of copper-related genes (**Figures 2A, B**). The PPI network (**Figure 2C**) and correlation analysis (**Figure 2D**) of copper-related genes in breast cancer samples showed the interactions between candidate genes. Genetic variation plays a crucial role in cancer origin and development. Therefore, we analyzed somatic mutations and CNV of copper-related genes in breast cancer samples (**Figures 2E, F**). According to the variant classification, the most prevalent variant, variant type and single nucleotide variant (SNV) were missense mutations, single-nucleotide polymorphisms (SNPs), and the C > T mutation, respectively. In breast cancer samples, ATP7A (18%), amyloid beta precursor protein (APP) (11%) and ATP7B (9%) were the more frequently mutated genes. Cuproptosis genes, such as dihydrolipoamide dehydrogenase (DLD) (2%) and dihydrolipoamide s-acetyltransferase (DLAT) (2%), were also among the top ten mutated genes.

### Construction of the breast cancer’s survival prediction model by copper-related genes

**Figure 3:**
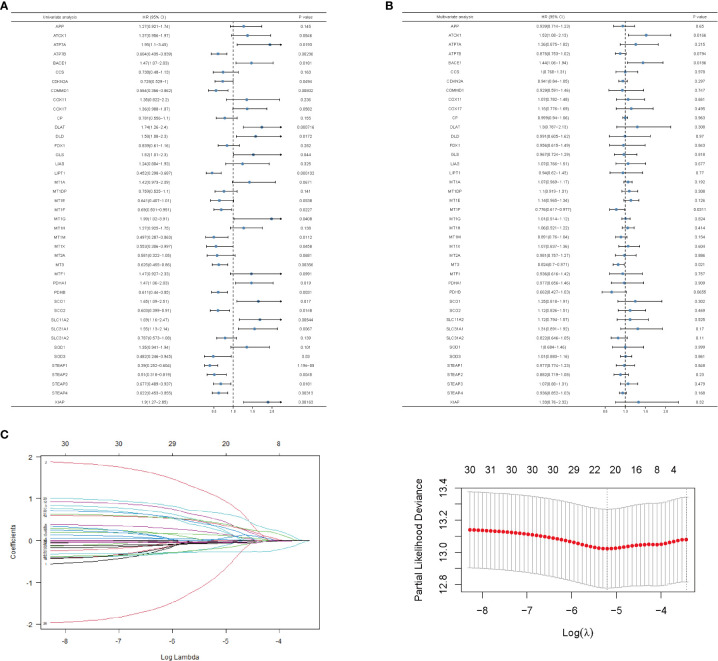
Univariate and multivariate analysis and LASSO-Cox regression of copper-related genes in breast cancer samples. Univariate **(A)**- and multivariate **(B)**-analysis of copper-related genes in breast cancer samples. **(C)** LASSO-Cox regression was built up from copper-related genes, based on which we selected optimal genes by the cross-validation method.

To predict the breast cancer survival pattern by a prognostic gene set, we utilized univariate and multivariate Cox regression analysis to plot the association between the expression of copper-related genes and the OS of breast cancer patients (**Figures 3A, B** and **Table S2**). Then, we built the LASSO-Cox model using univariate Cox regression genes (*p* value <0.1) to select the best candidate genes for constructing a survival prediction model of breast cancer patients (**Figure 3C**). Eventually, 21 candidate gene signatures emerged with the optimal log λ value of the LASSO-Cox model. We selected DLAT and ATP7B as the signature genes to construct the prediction model based on OS outcomes using regression coefficients. Risk score= 0.6664 x DLAT - 0.1985 x ATP7B.

### Prediction of breast cancer survival rates by gene expression of ATP7B and DLAT

**Figure 4:**
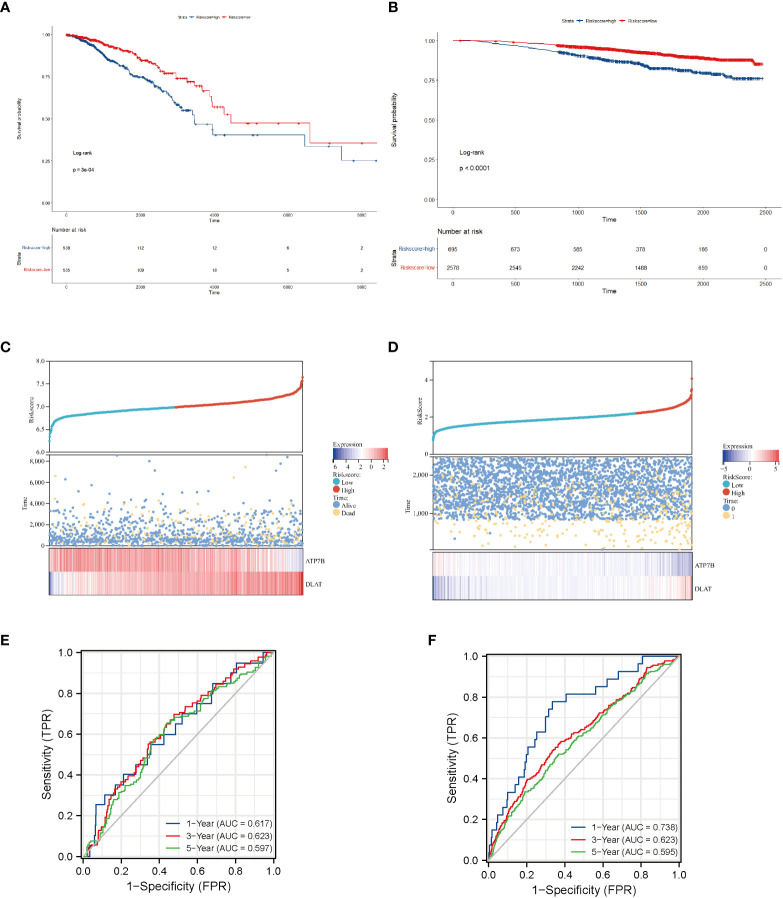
Survival analysis of breast cancer patients stratified by the risk score of copper-related genes. The Kaplan–Meier curves of TCGA-BRCA samples **(A)** and a validating dataset GSE96058 **(B)** grouped based on the risk score of copper-related genes at the best cut-off point. The statistical method is the Log-rank test. The low-scoring group had a better survival probability in both TCGA-BRCA samples and GSE96058 samples. The dot and line diagram of risk score, state of survival and expression of ATP7B and DLAT from TCGA-BRCA samples **(C)** and a validating dataset GSE96058 **(D)**. Time-dependent ROC curve of the constructed model of TCGA-BRCA samples **(E)** and the validating dataset **(F)**.

We confirmed the predictive performance of the prognostic gene set using the TCGA-BRCA dataset (**Figures 4A, C, E**) and a validating dataset (**Figures 4B, D, F**). **Figures 4A, B** presented Kaplan-Meier plot of the two risk groups’ OS in the training and validating dataset. We then further demonstrated the risk score distribution plot and expression of ATP7B and DLAT in breast cancer samples (**Figures 4C, D**). The survival plots indicated that the high- copper related genes scoring group had poor survival. For ease of description, we define the high- and low-copper related genes scoring groups as high- and low-scoring groups. Time-dependent ROC curves were constructed to evaluate the predictive model’s efficacy. At the 1-, 3-, and 5-year time points, the TCGA-BRCA dataset’s area under curves (AUCs) were 0.617, 0.623, and 0.597, respectively (**Figure 4E**). As for the validating breast cancer dataset (GSE96058), the areas under the time-dependent ROC curve were 0.738, 0.623 and 0.595 at the 1-, 3- and 5-year time points (**Figure 4F**).

### Comparison of the immune cells’ infiltration profile of the high- and low-scoring groups

**Figure 5:**
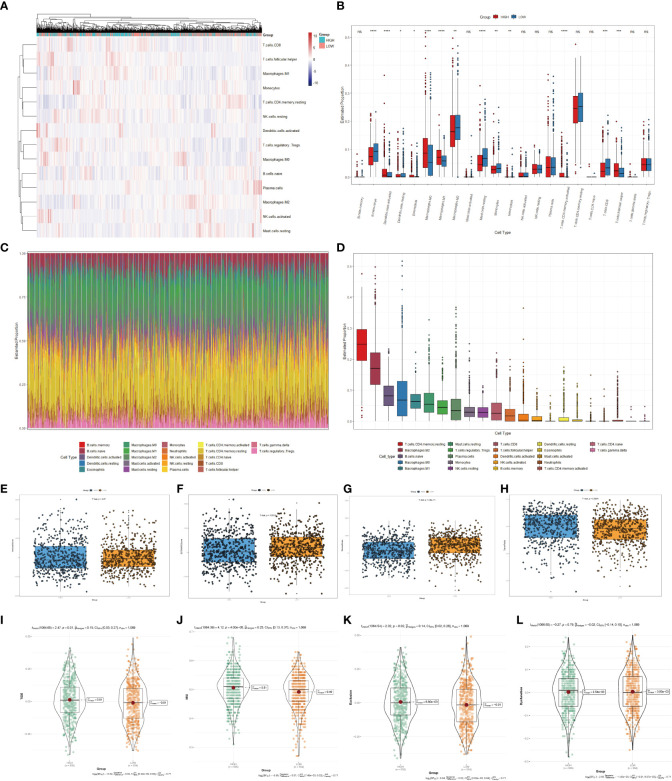
Immune cells infiltration analysis of the high- and low-scoring groups. Heatmap **(A)** and box plot **(B)** of immune cell abundance in breast cancer samples. **(C)** Histogram of the proportion of immune cells in each sample. **(D)** Box plot of the proportion of different immune cells (^*^p < 0.05; ^**^p < 0.01; ^***^p < 0.001, ^****^p< 0.0001). Box plots of the immune score (*p*=0.67) **(E)**, ESTIMATE score (*p* < 0.01) **(F)**, stromal score (*p*<0.0001) **(G)** and tumor purity (*p* < 0.01) **(H)** of the high- and low-scoring groups were calculated by ESTIMATE algorithm. Violin plots of Tide (*p*=0.01) **(I)**, MSI (*p <*0.0001) **(J)**, Exclusion (*p* =0.02) **(K)**, and Dysfunction (*p* =0.79) **(L)** of the high- and low-scoring groups were calculated by TIDE algorithm. (NS: no significance).

Immune infiltrates were increasingly considered responsible for influencing the prognosis and clinical outcome of breast cancer patients (32). Therefore, we compared the profile of tumor-infiltrating immune cells between the high- and low-scoring groups based on copper-related genes by heatmap (**Figure 5A**) and box plot (**Figure 5B**). The low-scoring group had more naive B cells, M2 macrophages, resting mast cells, monocytes, and CD8^+^ T cells than the high-scoring group, while the high-scoring group had more activated dendritic cells, M0 macrophages, M1 macrophages and follicular helper T cells. The histogram (**Figure 5C**) and box plot (**Figure 5D**) displayed the composition of different immune cells in breast cancer samples. In order to further estimate the immune statement of the two subgroups, four immune state indicators, including the Immune score (**Figure 5E**), ESTIMATE score (**Figure 5F**), stromal score (**Figure 5G**) and tumor purity (**Figure 5H**) were plotted. The result showed that the low-scoring group had a higher ESTIMATE score and stromal score and lower tumor purity. To assess the likelihood of immune evasion in tumors, we used TIDE to compare the gene expression profiles of the high- and low-scoring groups (33). The box plot of Tide, MSI, Exclusion, and Dysfunction (**Figures 5I–L**) also demonstrated that the low-scoring group had lower TIDE, Exclusion and MSI than those of the high-scoring group.

### Metabolic features of the high- and low-scoring groups

**Figure 6:**
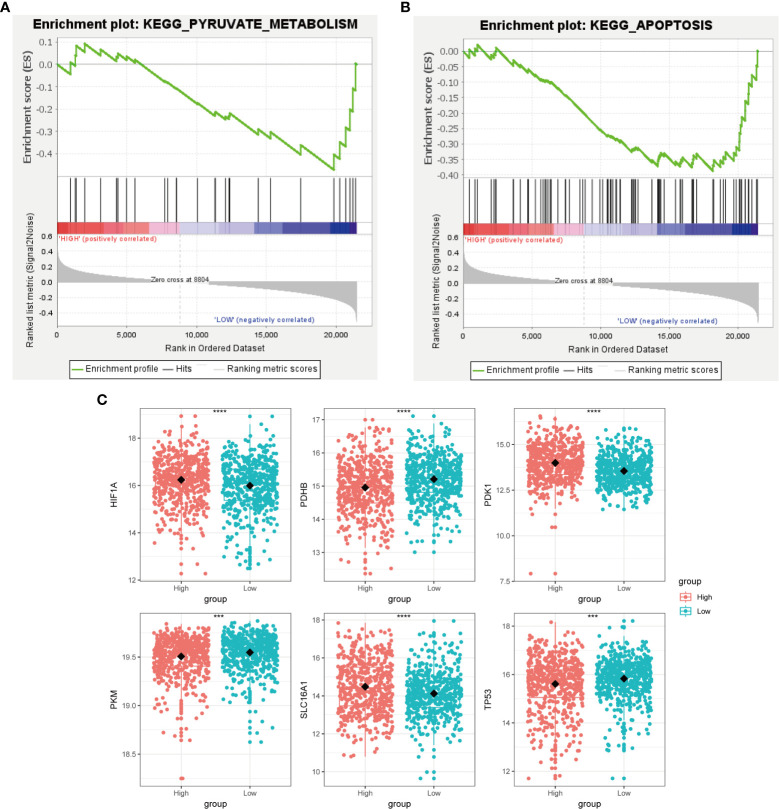
Metabolic characterization of breast cancer samples stratified by the high- and low-scoring groups. GSEA enrichment plot of regulation of autophagy **(A)** and pyruvate metabolism **(B)** of the low-scoring group. **(C)** Boxplot showed that glycolysis-related genes, including HIF1A, PDHB, PDK1, PKM, SLC16A1, and TP53, had a differential expression pattern among the high- and low-scoring groups. (^***^p < 0.001, ^****^p< 0.0001).

Cancer cells have a unique metabolic alteration known as aerobic glycolysis, in which glucose is preferentially converted to lactate even when oxygen is available (34). This phenomenon is in contrast to the typical cellular metabolism of non-malignant cells. GSEA demonstrated that breast cancer patients with lower scores for copper-related genes were more likely to have enrichment in pathways related to pyruvate metabolism and apoptosis (**Figures 6A, B**).

Tumor protein P53 (TP53), a crucial regulator of the Warburg effect, may influence glycolysis by reducing pyruvate dehydrogenase kinase-2 (Pdk2) expression, which results in the production of acetyl-CoA rather than lactate (35). We identified that the low-scoring group had a higher level of TP53 than the high-scoring group (**Figure 6C**). The pyruvate dehydrogenase (PDH) complex, which converts pyruvate to acetyl-CoA, controls pyruvate entering the citric acid cycle or participating in glycolysis. Pyruvate kinase M1/2 (PKM) converts phosphoenolpyruvate to pyruvate and can inhibit the expansion and metastasis of triple-negative breast cancer cells (36). We observed that the low-scoring group had a higher level of pyruvate dehydrogenase E1 subunit beta (PDHB) and PKM, which tends to produce pyruvate rather than lactate (**Figure 6C**). This result has revealed that the low-scoring group tended to rely on pyruvate metabolism for energy supply. Hypoxia inducible factor 1 subunit alpha (HIF1A) and the lactate transporter solute carrier family 16 member 1(SLC16A1) also regulate aerobic glycolysis in cancer metabolism, whose high expressions are correlated with poor clinical outcomes in breast cancer patients (37, 38). Pyruvate dehydrogenase kinase 1 (PDK1), a target of HIF1A, could prevent pyruvate from entering into the tricarboxylic acid cycle (TCA cycle) (39). The expression of HIF1A, SLC16A1 and PDK1 was increased in the high-scoring group (**Figure 6C**), suggesting its glycolysis metabolic feature.

### Treatment prognosis of the high- and low-scoring groups

**Figure 7:**
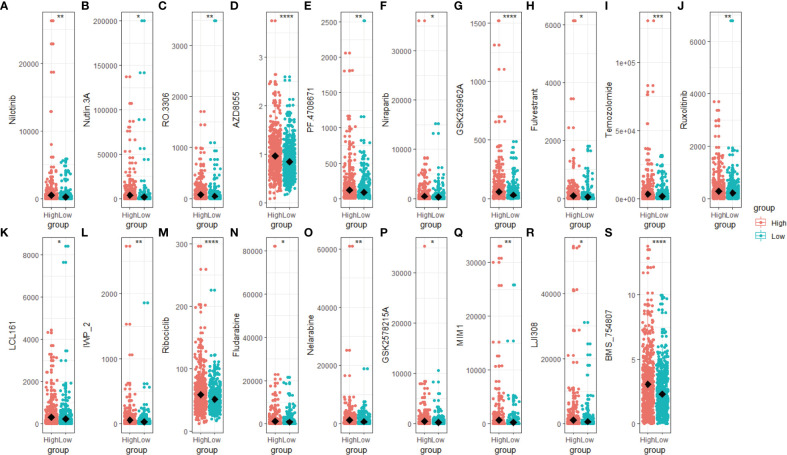
Drug sensitivity score of the high- and low-scoring groups. Box plot of the drug sensitivity score of Nilotinib **(A)**, Nutlin 3A **(B)**, RO 3306 **(C)**, AZD8055 **(D)**, PF4708671 **(E)**, Niraparib **(F)**, GSK269962A **(G)**, Fulvestrant **(H)**, Temozolomide **(I)**, Ruxolitinib **(J)**, LCL161 **(K)**, IWP_2 **(L)**, Ribociclib **(M)**, Fludarabine **(N)**, Nelarabine **(O)**, GSK2578215A **(P)**, MIM1 **(Q)**, LJI308 **(R)** and BMS_754807 **(S)**. The drug sensitivity score was predicted based on the R package “oncoPredict”, with a lower score representing a better clinical response. (^*^p < 0.05; ^**^p < 0.01; ^***^p < 0.001, ^****^p< 0.0001).

We predict breast cancer patients’ drug response using “oncoPredict”. The lower sensitivity score represented a more sensitive clinical response. Drugs with lower drug sensitivity scores in the low-scoring group were selected using the t-test (*p < 0.05)*. These selected drugs are Nilotinib, Nutlin 3A, RO 3306, AZD8055, PF4708671, Niraparib, GSK269962A, Fulvestrant, Temozolomide, Ruxolitinib, LCL161, IWP_2, Ribociclib, Fludarabine, Nelarabine, GSK2578215A, MIM1, LJI30 and BMS_754807 (**Figures 7A–S**). The low-scoring group had lower drug sensitivity scores than the high-scoring group, indicating that individuals in the low-scoring group responded better to the above-indicated chemotherapy drugs.

### ATP7B- and DLAT-related functional networks in breast cancer

**Figure 8:**
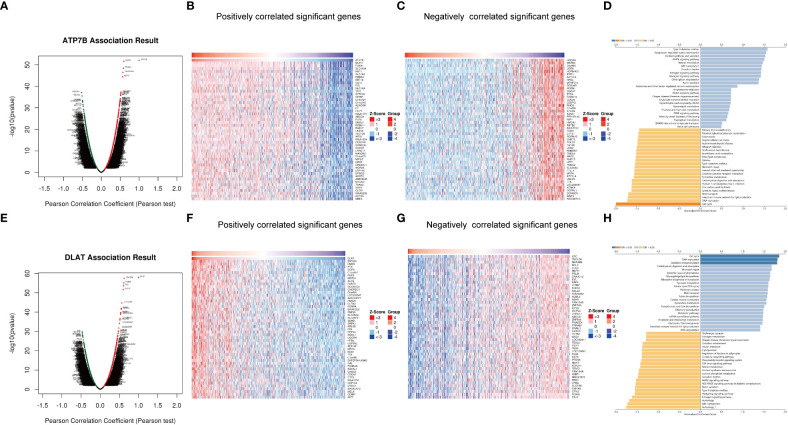
Genes co-expressed with ATP7B **(A–D)** and DLAT **(E–H)** in breast cancer. Volcano Plot showed genes associated with ATP7B **(A)** and DLAT **(E)** in breast cancer samples analyzed by LinkedOmics. Heatmap showed the positively correlated genes with ATP7B **(B)** and DLAT **(F)** and the negatively correlated genes with ATP7B **(C)** and DLAT **(G)** in breast cancer samples. The bar plot showed the GSEA results of genes associated with ATP7B **(D)** and DLAT **(H)**.

To reveal additional links to the biological function of ATP7B and DLAT in breast cancer development, we utilized the functional module of LinkedOmics to analyze genes that were positively or negatively correlated with ATP7B and DLAT (**Figures 8A–C, E–G**). Additionally, we performed an enrichment analysis on the association results (**Figures 8D, H**). ATP7B and its associated genes were enriched in the cell cycle pathway (*FDR* ≤ 0.05). DLAT and its associated genes were enriched in the cell cycle, oxidative phosphorylation and DNA replication pathways (*FDR* ≤ 0.05). The result of this study suggested that the two feature genes may contribute to the development of breast cancer by impacting cell growth and energy metabolism, potentially in collaboration with their co-expressed genes.

### Dysregulation of ATP7B and DLAT proteins in breast cancer

**Figure 9:**
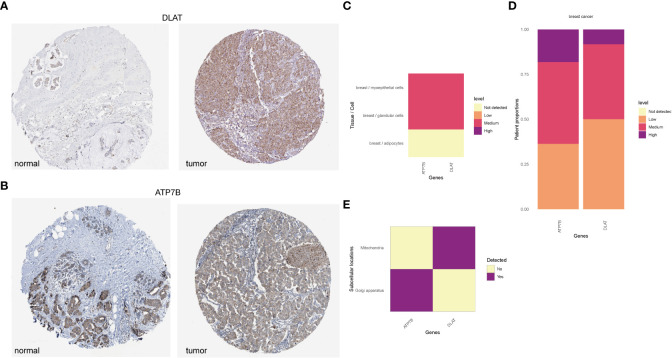
The protein expression of ATP7B and DLAT in BRCA tissues compared with non-tumor tissues in the HPA database. The protein expression of ATP7B **(A)** and DLAT **(B)** in breast cancer and normal tissues in the HPA database (http://www.proteinatlas.org) (40). **(C)** The expression of ATP7B and DLAT plotted according to cell types. **(D)** Column graphs showed the expression of ATP7B and DLAT in breast cancer samples. The subcellular localization of ATP7B and DLAT **(C–E)** was visualized by the R package “HPAanalyze” (41).

According to the HPA database (http://www.proteinatlas.org) (40), the high staining intensity of ATP7B and DLAT in breast cancer tissues is in contrast to those lowly stained in normal tissues as indicated by the immunohistochemical analyses (**Figures 9A, B**). HPAanalyze, a visualization R package, presented the expression of ATP7B and DLAT proteins in myoepithelial and glandular cells in breast cancer tissue using a heatmap (41) (**Figure 9C**). The IHC staining intensity of ATP7B and DLAT is shown in **Figure 9D**, and the subcellular locations of ATP7B (Golgi apparatus) and DLAT (mitochondria) are also indicated (**Figure 9E**).

### The expression profile and OS statement of different breast cancer subtypes

**Figure 10:**
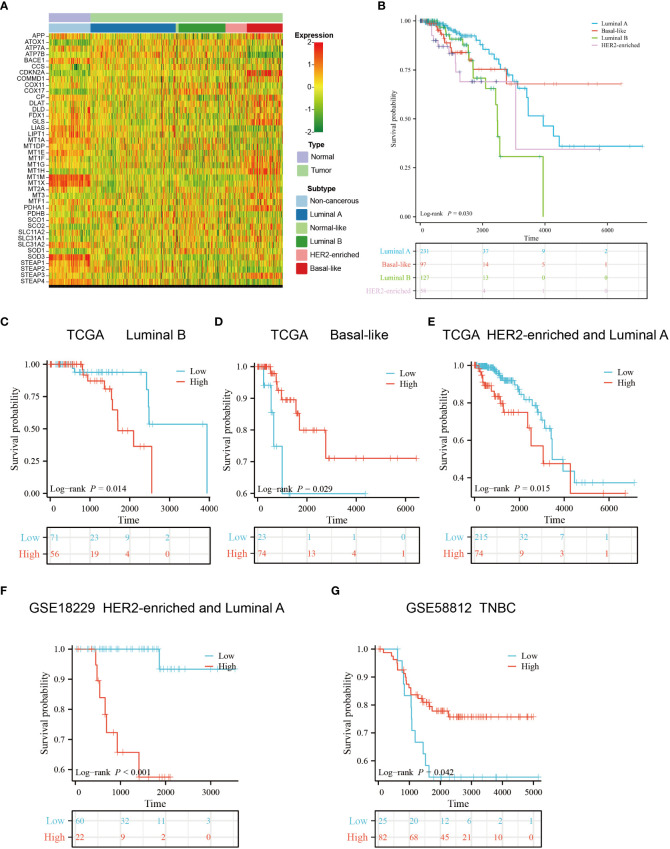
Gene expression profile and survival analysis of different subtypes of breast cancer stratified by the risk score of copper-related genes. **(A)** The gene expression heatmap of different subtypes of breast cancer. The subtype information was obtained from Xena. **(B)** The Kaplan–Meier curves of luminal A, luminal B, HER2-enriched and basal-like breast cancer patients. The Kaplan–Meier curves of luminal B **(C)**, basal-like **(D)**, luminal A and HER2-enriched patients **(E)** from TCGA. **(F)** The Kaplan–Meier curves of luminal A and HER2-enriched patients from GSE18229. **(G)** The Kaplan–Meier curves of Triple-negative breast cancers (TNBC) patients from GSE58812. The group was stratified based on the risk score of copper-related genes at the best cut-off point.

We obtained the subtype information of TCGA samples from XENA (42), based on which we grouped the primary breast cancers samples into five subtypes using the Prediction Analysis of Microarray 50 (PAM50) model, including luminal A, luminal B, normal-like, HER2-enriched and basal-like subtypes (43). The heatmap showed that copper-related genes had a differential expression pattern among breast cancer subtypes, indicating a potential role of copper in the heterogeneity of breast cancer (**Figure 10A**). Intriguingly, the expression of ATP7B and DLAT were decreased and increased respectively in the basal-like subtype compared with non-cancerous samples, which is opposite to those in other breast cancer subtypes. In addition to differences in copper-related gene expression, the survival status of breast cancer subtypes differed. The Kaplan–Meier curves of different breast cancer subtypes showed that the basal-like subtype had a worse survival probability than the luminal A- and luminal B-subtypes in the early stage (**Figure 10B**). We then used copper-related gene risk score to assess our predictive model in different subtypes. According to the survival curves, patients with basal-like subtype (**Figure 10D**) and triple-negative breast cancers (TNBC) (**Figure 10G**) present better survival in the high-scoring group and worse survival in the low-scoring group, in contrast to other subtypes (**Figures 10C, E, F**). This result suggests that the basal-like and TNBC patients had a unique copper-related genes profile among breast cancer subtypes.

### Copper staining of clinicopathological sections of breast cancer

**Figure 11:**
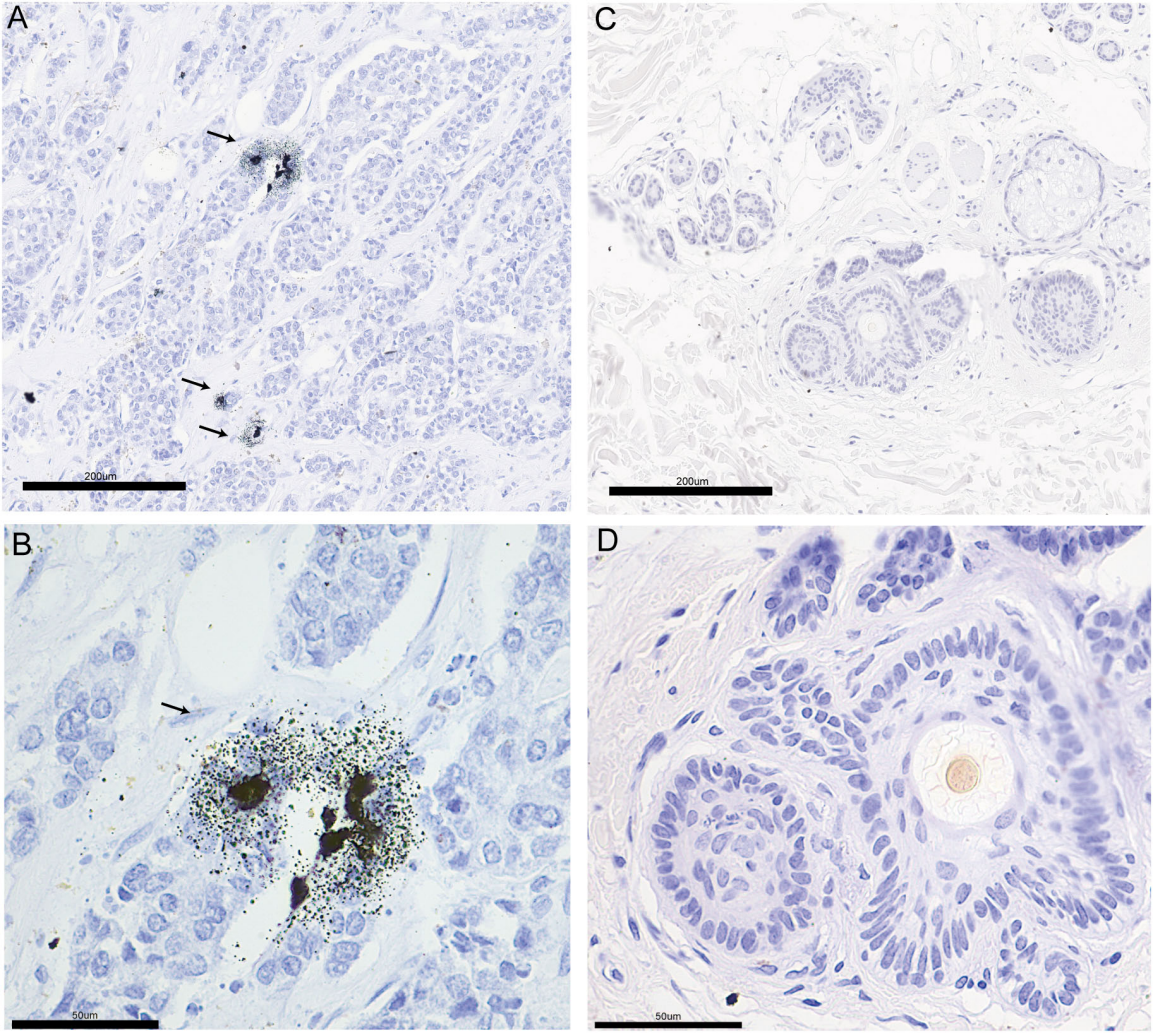
The copper stain of BRCA patients’ paraffin section using Timm’s method. Copper staining of the pathological section of breast cancer (**A**: 20x, **B**: 40x) and paired paracancerous (**C**: 20x, **D**: 40x) sample. The copper-positive areas contain small black granules. Coarse granules indicated intense copper deposition. The arrows indicate the distribution of copper in pathological sections.

According to literature reports, breast cancer patients have higher tissue and serum copper levels than normal subjects (44, 45). We performed Timms copper staining on the paraffin section of breast cancer patient to evaluate copper content and distribution in their tumor tissue. In the breast cancer sample, copper particles were found in the cytoplasm and nucleus of the breast cancer cells (**Figures 11A, B**). The paired paracancerous tissue did not yield a positive copper stain result (**Figures 11C, D**).

## Discussion

Breast cancer patients have been reported to exhibit higher serum and tissue content of copper, with even higher serum copper levels observed in patients non-responsive to chemotherapy (46–48). The amount of copper-containing cells was positively correlated with tumor growth rate (49). These results suggest that copper levels may indicate breast cancer progression and chemotherapy effectiveness in breast cancer patients. We found that copper particles in the clinical breast cancer sample were located in the cytoplasm and nucleus of the cancer cells (**Figures 11A, B**), which might be associated with the function of copper in promoting breast cancer metastasis. Several preclinical studies have found that reducing copper levels could inhibit tumor growth, angiogenesis and metastasis (50–52). Clinical trials using tetrathiomolybdate to deplete copper levels have enhanced event-free survival in breast cancer patients. Additionally, preclinical models have shown that tetrathiomolybdate could reduce breast cancer metastases to the lungs (53, 54). However, there is still a lack of elucidation on how copper content may influence breast cancer progression. Intriguingly, cuproptosis has been recently reported to mediate copper’s effect on cell death and cancer development. In breast cancer models, overloading copper by copper ionophores could inhibit tumor growth (55–57). These seemingly opposite conclusions prompted us to investigate the exact function of copper homeostasis in breast cancer development.

We constructed a copper-related gene scoring system using LASSO-Cox regression based on cuproptosis and copper metabolism genes to recognize the essential copper-related genes (**Figure 3C**). Two essential copper-related genes, ATP7B and DLAT, were selected to construct the scoring model to predict breast cancer patient survival. The higher AUCs of this model indicated advanced predictive performance (**Figure 4**). ATP7B, a P-type ATPase involved in copper secretion, played a pivotal role as a copper transporter, whose mutation caused Wilson’s disease due to excess copper accumulation-induced chronic liver diseases (58). DLAT, which is subjected to lipoylation modification, mediates the entry of carbon into the tricarboxylic acid cycle. Aggregation of lipoylated DLAT and reduction of iron-sulfur cluster proteins can be induced by copper ions, which results in proteotoxic stress and cell death (59). ATP7B and DLAT are both mutated in breast cancer samples, with the most common mutation being missense mutation (**Figures 2E–F**). Besides, we wonder what critical role ATP7B and DLAT played in breast cancer, given that these genes are essential for copper homeostasis and cuproptosis. The associated genes of ATP7B and DLAT genes are enriched in the cell cycle, oxidative phosphorylation, and DNA replication pathways (**Figures 8A–H**), suggesting that these two genes and their associated genes might influence breast cancer development by regulating the pathways mentioned above. Aerobic glycolysis, also known as the Warburg effect, is a characteristic metabolic process that is commonly observed in cancer cells (60). Many types of tumors limit the pyruvate oxidation process to meet the needs of the highly proliferative tumor cells (61). The low-scoring group is enriched in the pyruvate metabolism pathway (**Figure 6A**), suggesting that the low-scoring group might have an altered metabolic profile which is difficult to sustain the infinite growth of malignant cells. Breast cancer is heterogeneous in genetic and biological features (62). Generally, luminal A breast cancer had a better prognosis. Compared with the luminal A subtype, the luminal B-and HER2-enriched tumors present higher recurrence rates and worse survival (63, 64). The basal-like breast cancer is associated with poor prognosis, early relapses, and the highest locoregional recurrence among all subtypes (65, 66). Interestingly, basal-like patients had a unique expression and survival probability than other subtypes (**Figure 10**). The expression of ATP7B and SLC31A1 were decreased and increased, respectively, in the basal-like subtype patients (**Figure 10A**), suggesting that patients with the basal-like subtype of breast cancer may have different levels of copper in their tumor tissues compared with those with other breast cancer subtypes. This result might provide a comprehensive understanding of copper in different breast cancer subtypes.

Previous studies mainly focused on the relationship between cuproptosis-related genes and breast cancer (67, 68). Our study included not only cuproptosis-related genes but also copper metabolism-related genes to perform a comprehensive analysis of the role of copper-related genes in breast cancer development. Our results showed that the low-scoring group had lower expression of the copper importer SLC31A1 and higher expression of the copper exporter ATP7B (**Figures S1A, B**), which may altogether reduce intracellular copper content. The low-scoring group with less copper content appeared to have better survival outcomes and immune profiles. Combined with the evidence that copper chelators inhibited breast cancer metastasis, it is possible that reducing copper levels rather than increasing them is an effective way to improve breast cancer outcomes, which needs more experimental evidence for validation.

The composition of immune cells influences cancer progression. Evidence suggests that B cells are anti-tumor through various mechanisms, such as improving cytotoxic T cell activity and activating antibody dependence (69, 70). Activated CD8^+^ T lymphocytes are anti-tumor with cytotoxic molecules and have been reported to correlate with favorable prognosis in triple-negative breast cancer patients (71). In our result, the low-scoring group had more naive B cells and CD8^+^ T cells compared with the high-scoring group (**Figure 5B**), indicating better immune response in the low-scoring group. Additionally, because the copper chelate could reprogram and enhance the anti-tumor reaction of T cells (72), eliminating copper might be helpful for the anti-tumor response of breast cancer.

Based on the R package “oncoPredict”, we predict novel chemotherapy drugs which might be helpful for the low-scoring group’s breast cancer treatment. The low-scoring group seemed to be more responsive to chemotherapy drugs (**Figure 7**) which have been reported to suppress the metastasis or growth of breast cancer cells and overcome tamoxifen resistance by targeting essential regulators such as discoidin domain receptor 1, mTORC1/2, PARP-1/2, JAK1/2, and CDK1 (73–82). In the future, utilizing these newly developed chemotherapy drugs to treat breast cancer may be possible after conducting appropriate screening and classification and providing clinical guidance.

In summary, our study provided a novel prognostic signature to predict breast cancer development, which revealed the association of copper-related gene expression with immune cell infiltration, cancer metabolic feature, and drug response. These results may assist in the clinical management of breast cancer.

## Data availability statement

The original contributions presented in the study are included in the article/**Supplementary Material**. Further inquiries can be directed to the corresponding author.

## Ethics statement

The studies involving human participants were reviewed and approved by the multicenter clinical study on screening genetic mutation hotspots in Chinese breast cancer patients, Chinese PLA General Hospital. The patients/participants provided their written informed consent to participate in this study.

## Author contributions

MJ designed the study. YL and JW did data collection and analysis. YL and MJ wrote the manuscript. All authors contributed to the article and approved the submitted version.
