# Supplementary material for: Copper-related genes predict prognosis and characteristics of breast cancer
Source: Front Immunol. 2023 Apr 27;14:1145080. doi: 10.3389/fimmu.2023.1145080 (PMC10172490; doi:10.3389/fimmu.2023.1145080)
Supplement: Supplementary file 1 [file DataSheet_1.docx]

Supplementary Material

Copper-Related Genes Predict Prognosis and Characteristics of Breast Cancer


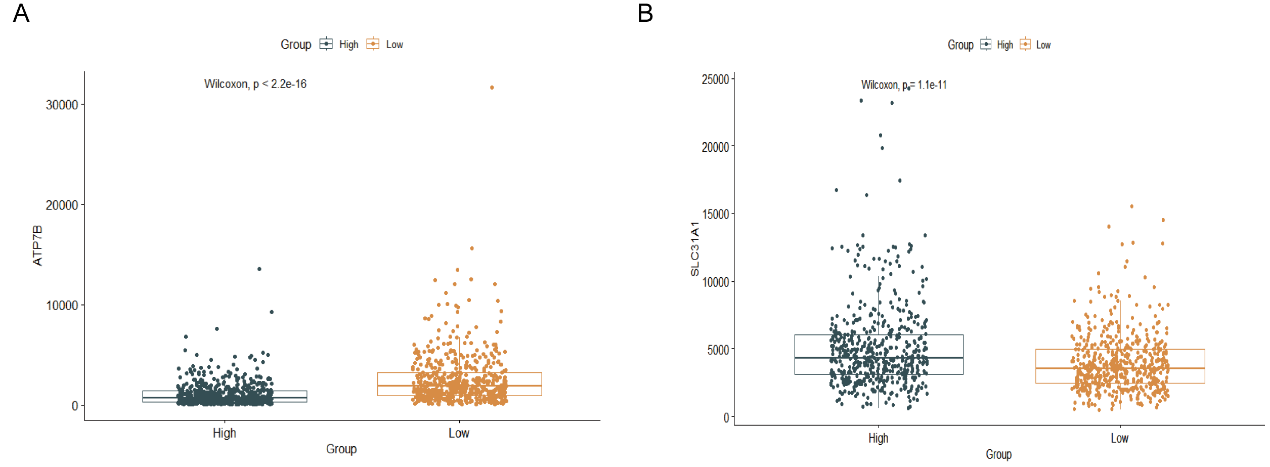


**Figure S1 The essential gene expression comparison of the high- and low-scoring groups.** The box plot expression of ATP7B (A) and SLC31A1 (B) in the high- and low-scoring groups.

**Table S1 Copper-related genes.**

| APP |
| --- |
| ATOX1 |
| ATP7A |
| ATP7B |
| BACE1 |
| CCS |
| CDKN2A |
| COMMD1 |
| COX11 |
| COX17 |
| CP |
| DLAT |
| DLD |
| FDX1 |
| GLS |
| LIAS |
| LIPT1 |
| MT1A |
| MT1DP |
| MT1E |
| MT1F |
| MT1G |
| MT1H |
| MT1M |
| MT1X |
| MT2A |
| MT3 |
| MTF1 |
| PDHA1 |
| PDHB |
| SCO1 |
| SCO2 |
| SLC11A2 |
| SLC31A1 |
| SLC31A2 |
| SOD1 |
| SOD3 |
| STEAP1 |
| STEAP2 |
| STEAP3 |
| STEAP4 |

**Table S2 Univariate and multivariate cox regression analysis of copper-related genes in breast cancer samples.**

|  | **Univariate** | | **Multivariate** | | |
| --- | --- | --- | --- | --- | --- |
| symbol | uni.HR.%95CI | uni.pvalue | mut.HR.%95CI | mut.pvalue | coef |
| APP | 1.27:(0.921-1.74) | 0.145 | 0.939:(0.714-1.23) | 0.65 | -0.0634 |
| ATOX1 | 1.37:(0.956-1.97) | 0.0846 | 1.52:(1.08-2.13) | 0.0166 | 0.416 |
| ATP7A | 1.95:(1.1-3.45) | 0.0193 | 1.26:(0.875-1.82) | 0.215 | 0.231 |
| ATP7B | 0.604:(0.435-0.839) | 0.00236 | 0.875:(0.753-1.02) | 0.0794 | -0.134 |
| BACE1 | 1.47:(1.07-2.03) | 0.0181 | 1.44:(1.06-1.94) | 0.0186 | 0.362 |
| CCS | 0.738:(0.48-1.13) | 0.163 | 1:(0.768-1.31) | 0.978 | 0.00386 |
| CDKN2A | 0.728:(0.529-1) | 0.0494 | 0.941:(0.84-1.05) | 0.297 | -0.0606 |
| COMMD1 | 0.554:(0.356-0.862) | 0.00802 | 0.929:(0.591-1.46) | 0.747 | -0.0742 |
| COX11 | 1.35:(0.822-2.2) | 0.236 | 1.07:(0.782-1.48) | 0.661 | 0.0711 |
| COX17 | 1.36:(0.988-1.87) | 0.0582 | 1.15:(0.776-1.69) | 0.495 | 0.136 |
| CP | 0.781:(0.556-1.1) | 0.155 | 0.999:(0.94-1.06) | 0.963 | -0.00141 |
| DLAT | 1.74:(1.26-2.4) | 0.000716 | 1.3:(0.787-2.13) | 0.308 | 0.259 |
| DLD | 1.58:(1.08-2.3) | 0.0172 | 0.991:(0.605-1.62) | 0.97 | -0.00946 |
| FDX1 | 0.839:(0.61-1.16) | 0.282 | 0.956:(0.615-1.49) | 0.843 | -0.0447 |
| GLS | 1.52:(1.01-2.3) | 0.044 | 0.967:(0.724-1.29) | 0.818 | -0.0339 |
| LIAS | 1.24:(0.804-1.93) | 0.325 | 1.07:(0.766-1.51) | 0.677 | 0.0718 |
| LIPT1 | 0.452:(0.298-0.687) | 0.000132 | 0.94:(0.62-1.43) | 0.77 | -0.062 |
| MT1A | 1.42:(0.973-2.09) | 0.0671 | 1.07:(0.969-1.17) | 0.192 | 0.0633 |
| MT1DP | 0.759:(0.525-1.1) | 0.141 | 1.1:(0.919-1.31) | 0.308 | 0.0915 |
| MT1E | 0.641:(0.407-1.01) | 0.0538 | 1.14:(0.965-1.34) | 0.126 | 0.128 |
| MT1F | 0.69:(0.501-0.951) | 0.0227 | 0.776:(0.617-0.977) | 0.0311 | -0.253 |
| MT1G | 1.99:(1.02-3.91) | 0.0408 | 1.01:(0.914-1.12) | 0.824 | 0.0114 |
| MT1H | 1.27:(0.925-1.75) | 0.138 | 1.06:(0.921-1.22) | 0.414 | 0.0586 |
| MT1M | 0.497:(0.287-0.863) | 0.0112 | 0.891:(0.76-1.04) | 0.154 | -0.115 |
| MT1X | 0.553:(0.306-0.997) | 0.0458 | 1.07:(0.837-1.36) | 0.604 | 0.0638 |
| MT2A | 0.581:(0.322-1.05) | 0.0681 | 0.981:(0.757-1.27) | 0.886 | -0.019 |
| MT3 | 0.625:(0.455-0.86) | 0.00356 | 0.824:(0.7-0.971) | 0.021 | -0.193 |
| MTF1 | 1.47:(0.927-2.33) | 0.0991 | 0.936:(0.616-1.42) | 0.757 | -0.0662 |
| PDHA1 | 1.47:(1.06-2.03) | 0.019 | 0.977:(0.656-1.46) | 0.909 | -0.0233 |
| PDHB | 0.611:(0.44-0.85) | 0.0031 | 0.662:(0.427-1.03) | 0.0655 | -0.413 |
| SCO1 | 1.65:(1.09-2.51) | 0.017 | 1.25:(0.818-1.91) | 0.302 | 0.223 |
| SCO2 | 0.603:(0.399-0.91) | 0.0148 | 1.12:(0.826-1.51) | 0.469 | 0.112 |
| SLC11A2 | 1.69:(1.16-2.47) | 0.00544 | 1.12:(0.794-1.57) | 0.525 | 0.111 |
| SLC31A1 | 1.55:(1.13-2.14) | 0.0067 | 1.31:(0.891-1.92) | 0.17 | 0.269 |
| SLC31A2 | 0.787:(0.573-1.08) | 0.139 | 0.822:(0.646-1.05) | 0.11 | -0.197 |
| SOD1 | 1.35:(0.941-1.94) | 0.101 | 1:(0.684-1.46) | 0.999 | -0.00029 |
| SOD3 | 0.482:(0.246-0.945) | 0.03 | 1.01:(0.883-1.16) | 0.861 | 0.0122 |
| STEAP1 | 0.39:(0.252-0.604) | 1.19E-05 | 0.977:(0.774-1.23) | 0.848 | -0.0229 |
| STEAP2 | 0.51:(0.318-0.819) | 0.0045 | 0.882:(0.719-1.08) | 0.23 | -0.125 |
| STEAP3 | 0.677:(0.489-0.937) | 0.0181 | 1.07:(0.88-1.31) | 0.479 | 0.0723 |
| STEAP4 | 0.622:(0.453-0.855) | 0.00313 | 0.936:(0.852-1.03) | 0.168 | -0.0661 |
| XIAP | 1.9:(1.27-2.85) | 0.00163 | 1.33:(0.76-2.32) | 0.32 | 0.283 |
